# Methylation of the *BIN1 *gene promoter CpG island associated with breast and prostate cancer

**DOI:** 10.1186/1477-3163-6-9

**Published:** 2007-05-04

**Authors:** Ekaterina B Kuznetsova, Tatiana V Kekeeva, Sergei S Larin, Valeria V Zemlyakova, Anastasiya V Khomyakova, Olga V Babenko, Marina V Nemtsova, Dmitry V Zaletayev, Vladimir V Strelnikov

**Affiliations:** 1 Research Centre for Medical Genetics, Russian Academy of Medical Sciences, Moscow, Russia; 2 Molecular Medicine Institute, I.M. Sechenov Moscow Medical Academy, Moscow, Russia; 3 Institute of Gene Biology, Russian Academy of Sciences, Moscow, Russia

## Abstract

**Background:**

Loss of BIN1 tumor suppressor expression is abundant in human cancer and its frequency exceeds that of genetic alterations, suggesting the role of epigenetic regulators (DNA methylation). *BIN1 *re-expression in the DU145 prostate cancer cell line after 5-aza-2'-deoxycytidine treatment was recently reported but no methylation of the *BIN1 *promoter CpG island was found in DU145.

**Methods:**

Methylation-sensitive arbitrarily-primed PCR was used to detect genomic loci abnormally methylated in breast cancer. *BIN1 *CpG island fragment was identified among the differentially methylated loci as a result of direct sequencing of the methylation-sensitive arbitrarily-primed PCR product and subsequent BLAST alliance. *BIN1 *CpG island cancer related methylation in breast and prostate cancers was confirmed by bisulphite sequencing and its methylation frequency was evaluated by methylation sensitive PCR. Loss of heterozygosity analysis of the BIN1 region was performed with two introgenic and one closely adjacent extragenic microsatellite markers.*BIN1 *expression was evaluated by real-time RT-PCR.

**Results:**

We have identified a 3'-part of *BIN1 *promoter CpG island among the genomic loci abnormally methylated in breast cancer. The fragment proved to be methylated in 18/99 (18%) and 4/46 (9%) breast and prostate tumors, correspondingly, as well as in MCF7 and T47D breast cancer cell lines, but was never methylated in normal tissues and lymphocytes as well as in DU145 and LNCaP prostate cancer cell lines. The 5'-part of the CpG island revealed no methylation in all samples tested. *BIN1 *expression losses were detected in MCF7 and T47D cells and were characteristic of primary breast tumors (10/13; 77%), while loss of heterozygosity was a rare event in tissue samples (2/22 informative cases; 9%) and was ruled out for MCF7.

**Conclusion:**

*BIN1 *promoter CpG island is composed of two parts differing drastically in the methylation patterns in cancer. This appears to be a common feature of cancer related genes and demands further functional significance exploration. Although we have found no evidence of the functional role of such a non-core methylation in *BIN1 *expression regulation, our data do not altogether rule this possibility out.

## Background

BIN1 (Bridging integrator 1) is a ubiquitous adaptor protein with the features of a tumor suppressor mediating apoptosis by c-MYC [1]. The *BIN1 *gene is located on chromosome 2q14 within a region reported to be deleted in 30% to 40% of breast carcinomas [2]. Complete or partial losses of BIN1 contained in this region in breast and prostate cancers have been reported [3,4]. Structural analysis of the human *BIN1 *gene [5] has promoted characterization of its molecular pathology in cancer. Notwithstanding the findings of genetic alterations, including allelic deletions, of *BIN1 *in breast cancer (BC), their frequencies are not sufficient to be responsible for all the cases of BIN1 losses occurring at the level of protein and/or message, suggesting a role for epigenetic factors [3]. As far as *BIN1 *promoter contains a CpG island [5], DNA methylation events that affect promoter activity offer a likely mechanism for epigenetic alteration in cancer. This suggestion is supported by the study of genes reactivation after treatment of prostate cancer cell lines with 5-aza-2'-deoxycytidine, in which reactivation of *BIN1 *was detected in the DU145 cells [6]. Although this finding suggests an epigenetic mechanism of *BIN1 *inactivation, bisulphite sequencing of the corresponding CGI had demonstrated "low" methylation. Methylation-specific PCR on the samples of primary tumors had not been performed [6]. By methylation-sensitive arbitrarily-primed PCR (MSe-AP-PCR) we have detected abnormal methylation of a fragment of *BIN1 *promoter region CGI in BC and studied its methylation patterns in primary breast and prostate cancer samples as well as in the MCF7 and T47D BC cell lines and DU145 and LNCaP prostate cancer cell lines.

## Methods

Ninety-nine paired (tumor/control) primary breast cancer and 46 primary prostate cancer samples were obtained from the Blokhin Cancer Research Center and Gertsen Moscow Oncology Research Institute and frozen at -70° until use. MCF7 cells were cultured in minimum essential medium (Eagle) with 2 mM L-glutamine, 10% fetal bovine serum (all from HyClone, USA), 0.1 mM non-essential amino acids, 1 mM sodium pyruvate, 100 u/ml penicillin, 100 ug/ml streptomycin, supplemented with 0.01 mg/ml bovine insulin (all from Invitrogen, USA). T47D cells were cultured in RPMI 1640 medium with 2 mM L-glutamine, 10% fetal bovine serum (all from HyClone, USA), 1.0 mM sodium pyruvate, supplemented with 0.2 Units/ml bovine insulin (all from Invitrogen, USA). DU145 cells were cultured in DMEM (Gibco, USA) supplemented with 10% fetal bovine serum (HyClone, USA). LNCaP cells were cultured in RPMI (Gibco, USA) supplemented with 10% fetal bovine serum (HyClone, USA) and 5% heat-inactivated horse fetal serum (Gibco, USA). After reaching the confluent cells were washed with D-PBS and detached by Trypsin-EDTA (HyClone, USA). Collected cells were used for DNA, RNA isolation.

**Figure 1:**
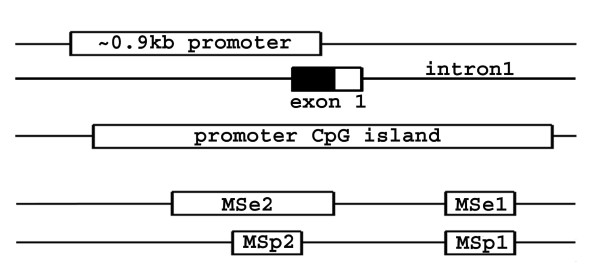
Structure of the *BIN1 *gene 5'-region and fragments of the BIN1 promoter CGI analyzed in this study, designated by the primer names (see table 1). 0.9 kb promoter corresponds to the region sufficient for basal transcription of BIN1 [5]. 5'-UTR is colored black.

DNA extraction was performed by standard phenol/chloroform method after proteinase K treatment of the tissues (cells). Screening for CpG islands differentially methylated in tumor and control sample pairs was performed by the methylation-sensitive arbitrarily-primed PCR (MSe-AP-PCR) described in [7] with the only exclusion that the primers were not radioactively labeled. MSe-AP-PCR fragments were run in nondenaturing polyacrylamide gels, stained by silver nitrate, excised, reamplified, and underwent direct sequencing on the ABI310 analyser (Applied Biosystems, USA) after HpaII digestion. Genomic locations of the differentially methylated fragments were identified by BLAST analysis and PCR primers directed to the corresponding regions were designed (table 1). Loss of heterozygosity (LOH) analysis of the BIN1 region was performed with two introgenic and one closely adjacent extragenic microsatellite markers (table 1). Methylation sensitive PCR (MSe-PCR) and methylation specific sequencing protocols were described previously [8]; corresponding primer sets used in this study are listed in table 1 and their relative positions are depicted in Fig. 1.

**Table 1:** Primers used in the study.

Primer designation	Primer sequence	Location	Type of analysis	Ann. t, C°	MgCl_2_, mM	Product size, bp
MSe1-F	tgctccaggctcttctcaggc	BIN1 promoter CGI, see Fig. 1	MSe PCR	61	1.5	255
MSe1-R	ggagaacgaaagtggagaagc					
MSe2-F	ggggctccgggcgcgttctcc			69	1.0	606
MSe2-R	actgcccatctctgccatcgc					
MSp1-F	gggaaaggaaatttaatttttttt		MSp sequencing	59	1.0	218
MSp1-R	aataataaccctcaactctccaaac					
MSp2-F	ggataataagttgttttttaaagggttatt			58	1.0	212
MSp2-R	ctccacctaatctcctttcctaaat					
BIN1-CA1F	cccgattttcattcaggtgtt	BIN1 intron 5	LOH	60	1.5	~210
BIN1-CA1R	agttccgagtttgatgccagc					
BIN1-CA2F	cttctacaccttccacaatca	3 kb distal to BIN1		60	1.5	~130
BIN1-CA2R	attcctttcctgtccctttgg					
BIN1-tccF	ccccaccccacaacctgaagt	BIN1 intron 1		60	1.5	~200
BIN1-tccR	tccctgtgtttcctggctaag					

RNA extraction was done with the Ribosole A kit (Interlabservice, Russia). Extracted total RNA underwent DNAse (Promega, USA) treatment at 37°C for 10 min with subsequent DNAse inactivation at 65°C for 5 min. Reverse transcription (RT) reaction was performed with the High Capacity cDNA Archive Kit (Applied Biosystems, USA). Real-time RT-PCR relative quantification of *BIN1 *expression in tumor/control pairs was performed on the ABI PRIZM 7000 SDS (Applied Biosystems, USA) with the provided TaqMan expression assays (BIN1, target, Hs00184913m1; 18s rRNA, endogenous control, Hs99999901s1; according to the Applied Biosystems TaqMan Gene Expression Assays nomenclature).

## Results

We have identified the *BIN1 *promoter CGI fragment among those differentially methylated in BC and morphologically intact tissues revealed by MSe-AP-PCR. After direct sequencing of the MSe-AP-PCR product MSe-PCR primers were designed and its methylation status in control and BC samples estimated. Methylation frequency in BC samples was determined as 18/99 (18%) for primary tumors; methylated status was also revealed in MCF7 and T47D cells. MSe analyses of prostate cancer samples resulted in the methylation figures of 4/46 (9%). No methylation was detected in control, morphologically intact tissues and lymphocytes as well as in DU145 and LNCaP prostate cancer cell lines.

**Figure 2:**
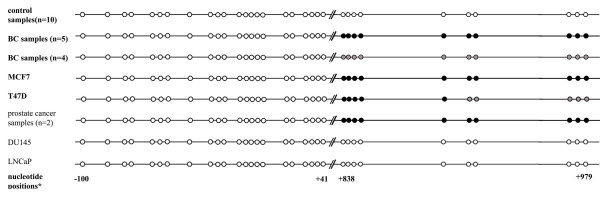
Distribution of methylated (black circles), unmethylated (white circles) and partially methylated (grey circles) CpG pairs across the promoter (left) and the first intron (right) parts of the BIN1 CpG island. *nucleotide positions were counted based on NCBI sequences NM_139343 and AC012508, position 1 corresponds to the 1^st ^mRNA nucleotide.

Methylation-specific sequencing of the corresponding fragment has allowed us to evaluate the methylation status of every CpG pair within the locus of interest and to construct the methylation maps for 9 BC and 2 prostate cancer samples that showed methylated status by MSe-PCR (fig. 2, positions from +838 to +979). Of these, 7 samples demonstrated biallelic patterns of methylation of all the CpGs contained within the fragment, while in the rest all the CpG pairs were partially methylated which can reflect either monoallelic status of methylation or contamination of the tumor samples with normal cells. Sequencing of 9 control samples, as well as of the DU145 and LNCaP cells revealed no methylated CpGs within the region. Analysis of MCF7 BC cells showed biallelic pattern of methylation of all CpG pairs within the sequenced fragment, while in the T47D BC cells 5 proximal CpG pairs demonstrated biallelic methylation and 5 distal CpG pairs were partially methylated. (fig. 2).

**Figure 3:**
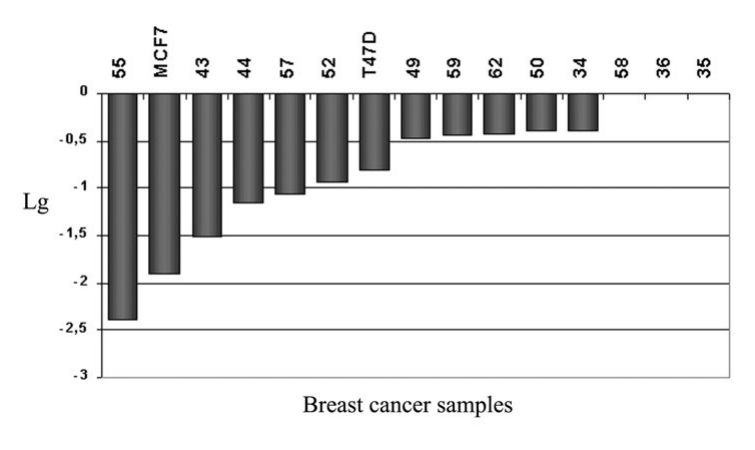
Expression of BIN1 in BC samples and cell lines measured by real-time RT-PCR. Relative expression is shown on a logarithm (lg) scale. Samples are numbered in accordance with our registry. MCF7 and T47D – BC cell lines.

*BIN1 *expression analysis revealed significant gene expression decrease/abrogation in 77% tumors (compared to apparently normal adjacent counterparts, 13 pairs tested), expression was completely arrested in the MCF7 cell line and 6-fold decreased in T47D compared to the control morphologically intact breast tissues (fig. 3). LOH analysis using two intragenic and one closely adjacent extragenic markers has excluded *BIN1 *deletion (LOH) in MCF7 cells by revealing the heterozygous state of the *BIN1 *intron 1 trinucleotide (tcc)_n _repeat and of the (ca)_n _repeat 3 kb distal to the gene. In T47D cells only the latter was informative and demonstrated heterozygous state with presumable partial LOH detected by decrease of band intensity corresponding to one of the alleles. Observed frequency of *BIN1 *LOH in primary breast tumors was unexpectedly low: 81% (22/27) were informative for at least one of the three microsatellite markers elaborated and only in 9% (2/22) of those allelic loss was detected.

As far as the fragment detected as abnormally methylated by MSe-AP-PCR is located within the 3' region (intron 1) of *BIN1 *promoter CGI (fig. 1) and its methylation would conventionally be considered not to affect gene expression, we have constructed primers directed to the 5'-end of the same CGI in order to evaluate methylation that may have possible functional consequences. By the results of both MSe-PCR (30 samples) and methylation-specific sequencing (25 samples) this part of the CGI appeared to be completely unmethylated in tumor samples, in MCF7, T47D, DU145, LNCaP cell lines and in 10 control samples.

## Discussion

Altered expression of the tumor suppressor BIN1 in various cancers had been previously reported in a number of papers. Sakamuro et al. reported significant reduction or complete loss of its expression in 14/27 BC cell lines including MCF7 and T47D as well as in 3/6 primary breast tumors by Northern analysis [1]. Complete or partial loss of BIN1 was reported in 6/6 estrogen receptor-positive and estrogen receptor-negative breast carcinoma cell lines and in 30/50 (60%) samples of malignant breast tissue analyzed by immuno-histochemistry or RT-PCR [3]. *BIN1 *mRNA was decreased in 25%, and lost in 67% of 24 breast cancer specimens in the study of Deng et al. [9]. RNA and immunohistochemical analyses indicate that *BIN1 *is expressed in most primary prostate tumors, even at slightly elevated levels relative to benign tissues, but that it is frequently missing or inactivated by aberrant splicing in metastatic tumors and androgen-independent tumor cell lines [4]. Genetic causes of *BIN1 *inactivation in prostate cancer mostly occur as a result of allelic losses [4]. The frequency of BIN1 losses exceeds the frequency of so far identified genetic alterations, suggesting a role for epigenetic factors [3]. One of these is *BIN1 *mRNA aberrant splicing detected in melanoma and prostate cancer [4,10]. Another likely mechanism for *BIN1 *epigenetic alteration might be presented by DNA methylation events that affect promoter activity [3]. The *BIN1 *promoter contains a CpG island [5]; nevertheless, we failed to find any report of a targeted methylation study of this region. Scarce information comes from the screening studies aimed to detect typical epigenetic alterations in cancer. Thus, Lodygin et al. report reactivation of BIN1 in one of the three prostate cancer cell lines, DU145, after 5-aza-2'-deoxycytidine treatment which suggested epigenetic regulation of the gene expression by promoter methylation. Based on the bisulphite sequencing methylation of *BIN1 *promoter in DU145 was classified as low, and methylation analyses in primary tumors have not been performed [6].

We have identified a fragment of *BIN1 *5'-CGI among those differentially methylated in BC by MSe-AP-PCR. Significant methylation levels of the corresponding region in breast and prostate primary tumors and its methylated status in MCF7 and T47D BC cells accompanied by a high frequency of decreased/abrogated expression of *BIN1 *in our cancer samples suggested possible involvement of methylation in *BIN1 *expression regulation.

Promoter CGIs often span more than 1 kilobase, and their methylation status is as a rule heterogeneous. Methylation often occurs in downstream regions of the promoter CGIs, but it is believed not to repress transcription [11,12]. Thus, the *CDKN2A *gene promoter CGI spans the promoter region and exon 1, but its transcription is only consistently repressed when a 230-bp region that covers the transcription start site is methylated [11]. *RASSF1A *also contains a promoter CGI that continues into its first exon, but methylation of a region covering the minimal promoter is enough for the loss of its expression [13]. *MLH1 *has a large promoter CGI that initiates at nucleotide -1,000 and extends throughout exon 1. Its methylation has been observed in far upstream regions, but only the methylation of a 280-bp region covering the transcription start site is correlated with transcription repression [14,15].

*BIN1 *promoter CGI spans 1650 bp of genomic sequence including 5'-upstream region, exon 1 and a fragment of intron 1 of the gene and obviously contains both promoter and nonpromoter sequences. Structural and functional analysis of a ~0.9 kb 5'-sequence adjacent to exon 1 had revealed that it contains consensus sites for several transcription factors and is sufficient for basal transcription of *BIN1 *[5]. As far as the promoter CGI fragment initially identified and analyzed in our study lies within intron 1 of the gene and does not belong to the promoter, to evaluate possible methylation of the genuine promoter region we have designed the sets of MSe and MSp primers directed to the corresponding locus. Surprisingly, but in consistence with the data presented by Lodygin et al. [6], we failed to detect its abnormal methylation even in the tumor samples methylated throughout the 3'-part of the promoter CGI. We suggest that *BIN1 *promoter CGI, similarly to those of *CDKN2A *or *RASSF1A *genes, is composed of the two functionally unequal parts, 3' being frequently and densely methylated and 5' being lowly methylated in cancer. This statement has two major concequences. On one side, as far as we have shown that the methylation of the 3'-part is cancer-specific, it can be elaborated in the development of epigenetic cancer markers after thorough evaluation of its methylation patterns in different types of tumors. On the other, the methylation analysis of the 3'-part may not be used in the functional studies (may not be taken as the evidence of expression loss) so far, as *a priori *assumption is that it is not likely to affect gene expression. It should be noted though, that the absence of such a functional link is to be supported by further exploration, which may altogether prove the opposite. Previously the analysis of more than 30 silenced genes and 100 non-silenced genes showed that methylation of only a relatively small 'core' region covering the transcription start site is consistently associated with gene silencing [16,17]. It has not yet been established, however, whether or not the core region should always contain the minimum promoter and the transcription start site [18].

## Conclusion

Our study supports previously obtained data of frequent cancer-associated loss of *BIN1 *expression in breast cancer. LOH and CpG methylation analyses demonstrate that these events, being among the most widespread causes of genes' inactivation in cancer, are nevertheless not responsible for the majority of cases of BIN1 loss in BC. Even in the case of functional significance of the *BIN1 *CGI 3'-part methylation, it would account for 18% of expression losses only, and the frequency of LOH in our BC samples is even lower. One possibility, which was never tested, is that BIN1 is downregulated in BC by alternative splicing like it is in prostate cancer and melanoma. Another one derives from the demethylation activation assay [6] and our methylation study on DU145 cells: reactivation of BIN1 expression after 5-aza-2'-deoxycytidine treatment in the cells with unmethylated status of its CGI suggests the role for the upstream epigenetically regulated factors.

## Abbreviations

BC – breast cancer; CGI – CpG island; MSe-PCR – methylation-sensitive PCR; MSe-AP-PCR – methylation-sensitive arbitrarily-primed PCR.
